# Do Maternal Factors Modify the Associations Between Iron Supplementation and Low Birth Weight in Sub‐Saharan Africa?

**DOI:** 10.1002/fsn3.70078

**Published:** 2025-04-03

**Authors:** Yibeltal Bekele, Don Vicendese, Melissa Buultjens, Mehak Batra, Bircan Erbas

**Affiliations:** ^1^ School of Psychology and Public Health La Trobe University Melbourne Victoria Australia; ^2^ School of Public Health Bahir Dar University Bahir Dar Ethiopia; ^3^ School of Population and Global Health The University of Melbourne Victoria Australia; ^4^ School of Computing, Engineering and Mathematical Sciences La Trobe University Melbourne Victoria Australia

**Keywords:** duration of supplementation, iron supplementation, low birth weight, sub‐Saharan countries

## Abstract

Iron supplementation is recommended to reduce low birth weight (LBW) but its impact in Africa is underexplored. This study examines factors that may modify the effects of maternal iron supplementation on LBW in sub‐Saharan Africa. Health Survey data from 26 sub‐Saharan countries, including 149,346 woman–infant pairs, were analyzed. LBW (< 2500 g) was the outcome, and iron supplementation (yes/no) and its duration (none, < 90 days, or ≥ 90 days) were exposures. A regression modeling framework was used to assess associations, adjusting for potential confounders and stratification by country income level. Family income, mother's education, maternal age, and partner's education were assessed as potential effect modifiers. The prevalence of LBW was 10.36%. Maternal iron supplementation adherence was 37.34%, but lower among poor and young women (31.43%). Not taking iron supplements during pregnancy increased the odds of LBW (aOR 1.19; 95%CI: 1.09, 1.30). Longer duration (more than 90 days) reduced the odds of LBW (aOR 0.84; 95%CI: 0.76, 0.93). These impacts were greater among poor women (aOR 0.74; 95%CI: 0.64, 0.84), women/partner with no education (aOR 0.79; 95%CI: 0.67, 0.92), and younger age (aOR 0.72; 95%CI: 0.54, 0.97). Taking iron supplements longer during pregnancy contributes to lowering LBW in sub‐Saharan countries. Younger mothers from poor areas with no education, along with those whose partners lack education, appear more vulnerable and may benefit from access to supplements. Enhancing adherence and addressing these disparities are key to addressing LBW in these settings.

## Graphical abstract

Iron supplementation during pregnancy reduces the odds of low birth weight (LBW), with longer supplementation showing greater protective effects. Extended iron supplementation benefits younger mothers, those from lower socioeconomic backgrounds, and women with limited education the most. Improving adherence to iron supplementation through targeted public health interventions is critical to addressing LBW in sub‐Saharan Africa.

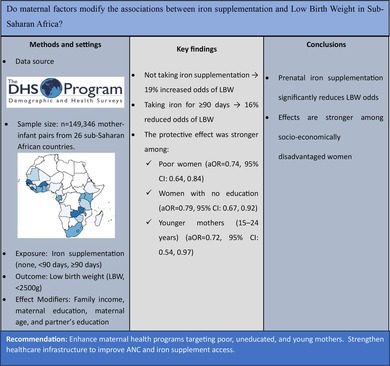

## 1 Introduction

Newborns with low birth weight (LBW), defined as less than 2500 g, face many challenges (Cutland et al. 2017). These fragile newborns experience morbidity and mortality rates over 10 times higher than those of normal‐weight infants (Mayor 2016). Additionally, they are more susceptible to chronic diseases like insulin‐resistant diabetes, hypertensive disorders, and cardiovascular disease later in life (Gluckman et al. 2008). The substantial economic burden is straining healthcare resources in high‐income and low‐income countries. For example, in the United States, annual healthcare costs for LBW infants reach USD (United States Dollar) 180 million, 37% of total infant healthcare costs, despite LBW infants being only 7% of all infants (Thanh et al. 2015). In sub‐Saharan Africa, the economic burden represents 10% of national per capita income (Mori et al. 2020). Reducing the prevalence of LBW in the region could avert up to 10% of national per capita income loss from treatment expenses and reallocate funds to other initiatives such as optimizing nutrition, enhancing health literacy, and improving living conditions. This, in turn, would reduce the disease burden on the child and the family.

In 2023, there were 19.8 million cases of LBW worldwide, comprising 14.7% of all live births. Over 91% occurred in low‐ and middle‐income countries, including 24% in sub‐Saharan Africa (Okwaraji et al. 2024). LBW is particularly challenging in sub‐Saharan Africa. For example, West Africa shows an LBW prevalence of 13.4% in Burkina Faso and 15.7% in Senegal (He et al. 2018). Similarly, a systematic review conducted in Ethiopia reported an LBW prevalence of 14.1% (Katiso et al. 2020), nearly double the prevalence observed in Western countries (ranging from 7.1% to 7.3%) (Okwaraji et al. 2024). This high prevalence highlights the significant public health challenge that LBW poses in these regions.

Despite various interventions to reduce LBW prevalence, in more than 20 years, the prevalence has only slightly decreased from 16.6% in 2000 to 14.7% in 2020 (Okwaraji et al. 2024). This is far below the 2.7% World Health Organization (WHO) target of reducing LBW by 30% by 2025, highlighting the inadequacy of current measures and the urgent need for more effective strategies (Okwaraji et al. 2024; World Health Organization (WHO) 2014). One such strategy could be addressing iron deficiency, which is vital for pregnancy and relies solely on diet (Percy et al. 2017). Deficiency might explain LBW (Figueiredo et al. 2018) through hormonal and oxidative stress mechanisms (Allen 2001). To combat iron deficiency, a significant issue for pregnant women in developing countries, the WHO promotes oral iron supplementation (World Health Organization (WHO) 2016). Despite the benefits of this supplement for birth outcomes, adherence in sub‐Saharan countries remains low, averaging 28.7% (with a range of 1.4% to 73%). This situation is particularly alarming, as 36.4% of the countries in the region report adherence rates below 25% (Ba et al. 2019).

Significant disparities exist within the region, with adherence likely lower in low‐income countries (Ba et al. 2019). Understanding the implications of these variations by country income and demographics is crucial for designing and implementing targeted interventions to improve adherence and ensure all pregnant women can benefit from iron supplementation.

While some studies in this region support iron supplementation for reducing LBW, they often have limitations. Our recent systematic review and meta‐analysis conducted in Africa showed that iron supplementation significantly reduced the odds of LBW. However, the review highlighted several gaps, including small sample sizes varying by country, inadequate control of confounders, insufficient data on the duration of supplementation, and a lack of targeted analysis for vulnerable populations with healthcare access disparities. The review recommended further regional research to address these gaps (Bekele et al. 2024). By addressing previous methodological shortcomings, this cross‐sectional study aims to provide more comprehensive evidence on the benefits of oral iron supplements, including what role longer duration of maternal iron supplementation has on LBW. Additionally, it seeks to identify key sociodemographic factors that may modify the effects. The findings will provide a deeper understanding of the combined impacts of maternal, family, and income factors with iron supplementation, which can then be used to inform policy and guidelines.

## 2 Methods

### 2.1 Data Source and Study Population

The Demographic Health Survey (DHS) program, initiated by the United States Agency for International Development (USAID), aims to provide data for informed decision‐making (Wang et al. 2021). Covering over 90 countries in Africa, Asia, Latin America/Caribbean, and Eastern Europe, it includes 35 countries in sub‐Saharan Africa. The DHS program collects data every 5 years from each country on fertility, family planning, reproductive health, child health, nutrition, HIV/AIDS, and malaria through community‐based, nationwide surveys in collaboration with country governments and the Inner‐City Fund (ICF) International. Further details about the program can be found on the DHS webpage (https://dhsprogram.com/). The ethical review of the survey was approved by both the ICF Institutional Review Board (IRB) and the IRB of the host country. Further approval was obtained from La Trobe University with an ethical application number HEC23324.

**FIGURE 1:**
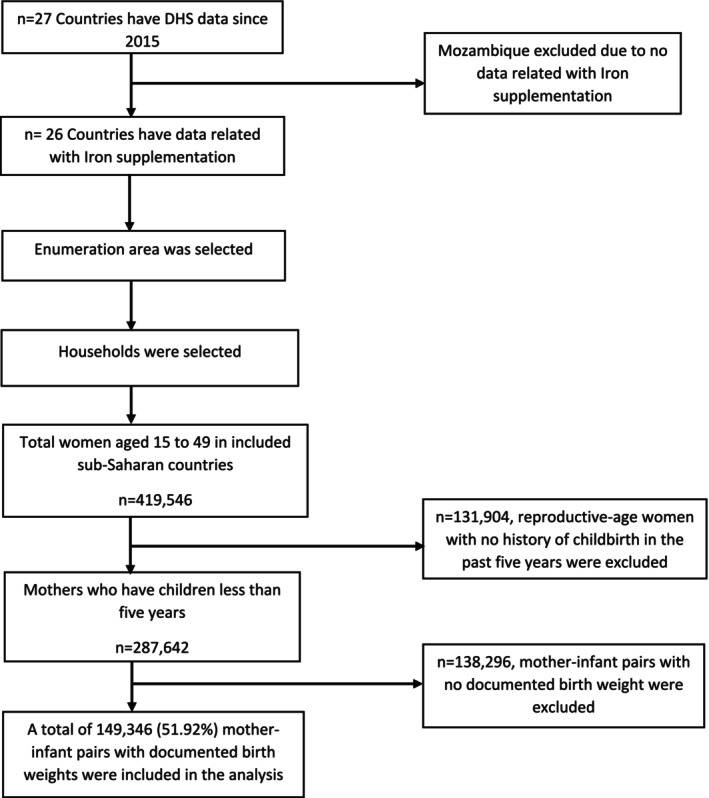
Flow diagram of the sampling procedure followed by DHS and participants included in the analysis.

This study used a two‐stage stratified random sampling technique to analyze DHS data from 2015/16 to 2022/23 in sub‐Saharan Africa. Rural and urban residences were initially categorized, and enumeration areas (EA) were selected proportional to EA size. Households within selected EAs were then systematically included in the survey. Data from 26 sub‐Saharan countries, including Angola, Benin, Ethiopia, Ghana, Kenya, Nigeria, and Uganda, cover 287,642 newborns. This analysis included 149,346 (51.92%) of the 287,642 mother‐infant pairs with documented birth weight (Figure 1).

### 2.2 Data Collection Methodology

Data was collected once after having an infant within 5 years. Mothers with children under five were asked about their socioeconomic status, pregnancy experiences, and the prenatal services they received. Data were collected via maternal recall using standardized questionnaires translated into local languages. Qualified data collectors and supervisors proficient in the local language were recruited and underwent rigorous training lasting 3–4 weeks. This training included mock interviews, demonstrative interviews, and actual field interviews focused on understanding the survey objective, mastering data collection tools, and ensuring accurate data capture. The survey implemented rigorous multilevel supervision mechanisms to uphold data quality. This included daily oversight by field supervisors and editors and regular visits to field sites by central office staff (ICF International 2012).

### 2.3 Outcome

Low birth weight (< 2500 g) was the primary outcome. Newborns were categorized as having low birth weight (“yes”) or not (“no”) based on weight (grams) records documented in health cards verified by trained data collectors. Women without health cards were excluded due to missing data.

### 2.4 Exposures

Iron supplementation (yes/no) was assessed by asking women, “During this pregnancy, were you given, or did you buy any iron tablets or iron syrup?” Duration of supplementation was categorized as no, less than 90 days, or 90 days or more, based on the question, “During the whole pregnancy, for how many days did you take the tablets or syrup?”. To account for variations in packaging and brands across different regions in sub‐Saharan Africa, data collectors presented sample iron tablets commonly used in the specified community to participants unsure about their supplementation. While the brands of iron tablets used in each country were not specified, this approach aimed to improve recall accuracy. However, it is important to note that access to and familiarity with iron supplements may vary, particularly in remote areas.

### 2.5 Other Variables

A comprehensive literature search identified factors that could contribute to the pathway from iron supplementation to the risk of low birth weight. A directed acyclic graph (DAG) was used to identify potential confounding, adjustment, and effect modification variables. Some of these variables were antenatal care utilization (yes or no) and maternal age at the birth of offspring (details attached as a method File S1). The DAG was drawn using online browsers (Textor et al. 2016) (Figure S1).

### 2.6 Statistical Analysis

Descriptive statistics summarized the distributions of exposure variables, the outcome variable, and other variables, with differences tested using the chi‐square test, as all variables were categorical. The DHS program uses a two‐stage cluster sampling technique, resulting in a hierarchical data structure that violates the regression assumption of independence of observations. This is because study subjects are nested within households and households within enumeration areas (EAs), where women might share similar characteristics. Therefore, a Generalized Linear Mixed Model (GLMM) was used to analyze the association between iron supplementation and low birth weight, incorporating random effects to account for data non‐independence. The outcome, birth weight, was dichotomized into 1 = yes (less than 2500 g) and 0 = no (2500 g or more).

Models were stratified by countries' income levels, with sub‐Saharan countries classified into low‐income and lower‐middle‐income categories according to the World Bank 2022 classification (World Bank 2024). Low‐income countries included Burundi, Burkina Faso, Ethiopia, Gambia, Liberia, Madagascar, Malawi, Mali, Rwanda, Sierra Leone, and Uganda. Lower‐middle‐income countries included Angola, Benin, Cameroon, Cote d'Ivoire, Gabon, Ghana, Guinea, Kenya, Mauritania, Nigeria, Senegal, South Africa, Tanzania, Zambia, and Zimbabwe.

To assess modifiers of these associations, we further stratified the analysis by family income (low‐income or high), maternal education (lower educational status and higher educational status), maternal age (15–24 years), and partner education (higher educational status or no education). We included the younger age groups in the stratification analysis due to their heightened risk of complications. Adjusted odds ratios with 95% confidence intervals (CIs) estimated the effects of iron supplementation and its duration on LBW. Statistical analyses were performed using STATA 18 (StataCorp 2023), with a significance level of *p* ≤ 0.05. For stratification analysis, we focused on trends and *p* values of < 0.1 to ensure that we did not miss any important effects due to the small sample size in certain strata.

## 3 Results

### 3.1 Descriptive Statistics of the Participant

Characteristics of the sample by low and lower‐middle income are summarized in Table 1. The prevalence of LBW was 10.36% (95% CI: 10.32, 10.62) in sub‐Saharan Africa (ranging from 10.27% in low‐income and 10.43% in lower‐middle‐income countries). Adherence to iron supplementation (≥ 90 days) in the region was 37.34% (95% CI: 36.52, 38.04), with significant variations by income level: 44.27% in lower‐middle‐income countries and 27.93% in low‐income countries (*p* < 0.001). Mothers who did not take iron supplements had a higher percentage of LBW babies (11.22%, *n* = 1547) compared to those who did (9.56%, *n* = 9689; *p* < 0.001). Similar trends were observed across both income groups (Table 1).

**TABLE 1:** Descriptive characteristics of the participants in sub‐Saharan countries.

Characteristics		sub‐Saharan countries (*n*=149346), (Low birth weight)		Low‐income (*n*=72,107), (Low birth weight)		Lower‐middle‐income (*n*=77,239), (Low birth weight)	
		Yes (%)	No (%)	Yes (%)	No (%)	Yes (%)	No (%)
Iron supplementation* *n* = 114,790	Yes	9689 (9.56)	91,311 (90.41)	4432 (9.50)	42,216 (90.50)	5,257 (9.67)	49,094 (90.33)
	No	1547 (11.22)	12,245 (88.78)	879 (10.70)	7336 (89.30)	668 (11.27)	4908 (88.02)
Duration of iron supplementation* *n* = 108,213	No	1547 (11.22)	12,245 (88.78)	879 (10.70)	7336 (89.30)	668 (11.98)	4909 (88.02)
	< 90 days	4869 (10.29)	42,466 (89.71)	2718 (9.93)	24,653 (90.07)	2151 (39.08)	17,813 (89.23)
	≥ 90 days	4196 (8.91)	42,890 (91.09)	1511 (9.02)	15,245 (90.98)	2685 (8.85)	27,644 (91.15)
Age* *n* = 149346	15–19	1350 (14.64)	7870 (85.36)	632 (15.45)	3456 (84.55)	718 (14.00)	4412 (86.00)
	20–24	4129 (11.89)	30,612 (88.11)	1989 (11.72)	14,976 (88.28)	2140 (12.04)	15,635 (87.96)
	25–29	3914 (9.94)	35,460 (90.06)	1797 (9.63)	16,861 (90.37)	2117 (10.22)	18,599 (89.78)
	30–34	2885 (8.99)	29,203 (91.01)	1366 (8.80)	14,162 (91.20)	1519 (9.17)	15,041 (90.83)
	35–39	2098 (9.53)	19,922 (90.47)	1037 (9.51)	9864 (90.49)	1061 (9.54)	10,058 (90.46)
	40–44	968 (10.24)	8487 (89.76)	475 (10.14)	4208 (89.86)	493 (10.33)	4279 (89.67)
	45–49	242 (9.89)	2206 (90.11)	112 (8.74)	1170 (91.26)	130 (11.15)	1036 (88.85)
Educational status* *n* = 149346	Illiterate	4554 (11.53)	34,954 (88.47)	2334 (10.90)	19,081 (89.10)	2220 (12.27)	15,874 (87.73)
	Primary	5479 (10.54)	46,508 (89.46)	3330 (10.72)	27,744 (89.28)	2149 (10.28)	18,764 (89.72)
	Secondary	4863 (9.92)	44,147 (90.08)	1544 (9.17)	15,289 (90.83)	3319 (10.31)	28,858 (89.69)
	Higher	690 (7.80)	8151 (92.20)	200 (7.18)	2586 (92.82)	490 (8.09)	5565 (91.91)
Wealth index* *n* = 149346	Poorest	3365 (12.00)	24,688 (88.00)	1792 (11.81)	13,386 (88.19)	1573 (12.22)	11,302 (87.78)
	Poorer	3196 (11.08)	25,639 (88.92)	1493 (10.60)	12,590 (89.40)	1703 (11.54)	13,049 (88.46)
	Middle	3209 (10.51)	27,324 (89.49)	1466 (10.65)	12,300 (89.35)	1743 (10.40)	15,024 (89.60)
	Richer	3081 (10.05)	27,575 (89.95)	1345 (9.89)	12,260 (90.11)	1736 (10.18)	15,315 (89.82)
	Richest	2735 (8.75)	28,534 (91.25)	1312 (8.48)	14,163 (91.52)	1423 (9.01)	14,371 (90.99)
Maternal working* *n* = 149346	Yes	9363 (9.68)	87,363 (90.32)	5012 (9.76)	46,333 (90.24)	4351 (9.59)	41,030 (90.14)
	No	6223 (11.83)	46,397 (88.17)	2396 (11.54)	18,366 (88.46)	3827 (12.01)	28,031 (87.99)
Marital status* *n* = 149346	Single	1705 (12.92)	11,496 (87.08)	458 (11.46)	3539 (88.54)	1247 (13.55)	7957 (86.45)
	Married	9854 (10.00)	88,728 (90.00)	5039 (10.04)	45,151 (89.96)	4815 (9.95)	43,577 (90.05)
	Living separate	2809 (10.51)	23,921 (89.49)	1255 (10.15)	11,104 (89.85)	1554 (10.81)	12,817 (89.19)
	Widowed	162 (9.24)	1592 (90.76)	104 (10.92)	848 (89.08)	58 (7.23)	744 (92.77)
	Divorce	347 (12.39)	2454 (87.61)	187 (14.22)	1128 (85.78)	160 (10.77)	1326 (89.23)
	Separated	709 (11.29)	5569 (88.71)	365 (11.08)	2929 (88.92)	344 (11.53)	2640 (88.47)
ANC* *N* = 115,049	Yes	10,957 (9.79)	101,979 (90.30)	5211 (9.59)	49,116 (90.41)	5746 (9.80)	52,863 (90.20)
	No	310 (14.67)	1803 (85.33)	111 (18.29)	496 (81.71)	199 (13.21)	1307 (86.79)
Parity* *n* = 149346	Nullipara	3379 (12.06)	24,631 (87.94)	1596 (12.38)	11,291 (87.62)	1783 (11.79)	13,340 (88.21)
	Primipara	3567 (10.82)	29,387 (89.18)	1619 (10.53)	13,762 (89.47)	1948 (11.09)	15,625 (88.91)
	Multipara	6026 (9.59)	56,783 (90.41)	2853 (9.40)	27,492 (90.60)	3173 (9.77)	29,291 (90.23)
	Grand multipara	2614 (10.22)	22,959 (89.78)	1340 (9.93)	12,154 (90.07)	1274 (10.55)	10,805 (89.45)
Media exposure* *n* = 149346	No	5157 (11.29)	40510 (88.71)	2720 (11.21)	21,546 (88.79)	2437 (11.39)	18,964 (88.61)
	Yes	10,429 (10.06)	93,250 (89.94)	4688 (9.80)	43,153 (90.20)	5,741 (10.28)	50,097 (89.72)
Accessing health care* n=149346	Not a big problem	6112 (9.80)	56,279 (90.20)	2560 (9.41)	24,632 (90.59)	3552 (10.09)	31,647 (89.91)
	A big problem	9474 (10.90)	77,481 (89.10)	4848 (10.79)	40,067 (89.21)	4626 (11.00)	37, 414 (89.00)
Partner Education* n=125,238	Illiterate	3804 (11.42)	29,506 (88.58)	1996 (10.62)	16,805 (89.38)	1808 (12.46)	12,701 (87.54)
	Primary	3831 (9.98)	34,567 (90.02)	2467 (10.27)	21,559 (89.73)	1364 (9.49)	13,008 (90.51)
	Secondary	3966 (9.77)	36,630 (90.23)	1480 (9.81)	13,607 (90.19)	2486 (9.75)	23,023 (90.25)
	Higher	1052 (8.31)	11,885 (91.87)	351 (7.57)	4284 (92.43)	701 (8.44)	7601 (91.56)

*Note:* Chi‐square test was used; ANC: antenatal care. *p = < 0.001.

The prevalence of LBW varied significantly across various demographic factors. The highest prevalence was among mothers with no formal education (11.53%, *n* = 4554), the poorest households (12.00%, *n* = 3365), and women without antenatal care (14.67%, *n* = 310). Partner education also influenced LBW prevalence, with the highest percentage among those with no formal education (11.42%, *n* = 3804), decreasing with higher education levels (*p* < 0.001). These patterns were consistent within all income groups (Table 1).

Overall, in a crude analysis, sub‐Saharan countries and lower‐middle‐income countries not taking iron supplementation during pregnancy showed a significant association with an increased likelihood of LBW compared to those who did [(crude odds ratio (cOR) 1.11; 95% CI: 1.04, 1.18; *p* < 0.001) and (cOR 1.09; 95% CI: 1.01, 1.25; *p* < 0.001) respectively]. When pregnant women took iron supplements for at least 90 days, they had overall lower odds of LBW babies than those who did not take the supplements (cOR 0.83; 95% CI: 0.77, 0.89; *p* < 0.001). This finding is consistent across all income groups (Table S1).

In the adjusted models, not taking oral iron supplementation remained statistically significant in increasing the odds of low birth weight (aOR 1.19; 95% CI: 1.09, 1.30; *p* < 0.001) compared to those who did take the supplement, and this pattern was consistent within each income group. Taking iron supplements for at least 90 days significantly lowered LBW odds (all: aOR 0.84; 95%CI: 0.76, 0.93; *p* < 0.001, low‐income: aOR 0.88, 95%CI: 0.78, 0.99; *p* = 0.04, lower‐middle‐income: aOR 0.82; 95%CI: 0.71, 0.94; *p* < 0.01) (Table 2).

**TABLE 2:** Adjusted analysis of iron supplementation and its duration on low birth weight in sub‐Saharan countries.

Exposures		Sub‐Saharan countries aOR (95%CI)	*p*	Low income aOR (95%CI)	*p*	Lower‐middle income aOR (95%CI)	*p*
Iron supplementation^#^	Yes	1	< 0.001*	1	0.04*	1	0.01*
	No	1.19 (1.09, 1.30)		1.13 (1.01, 1.28)		1.22 (1.06, 1.41)	
	≥ 90 days	0.84 (0.76, 0.93)	< 0.001*	0.88 (0.78, 0.99)	0.04*	0.82 (0.71, 0.94)	0.01*
Duration of iron supplementation	< 90 days	1.01 (0.93, 1.10)	0.83	1.00 (0.89, 1.12)	0.99	1.03 (0.89, 1.18)	0.69
	No	1		1		1	

*Note:* Educational status of the mother, partner's educational status, age of the mother, media exposure, wealth index, access to care, parity, antenatal care service utilization, and residence were controlled; ^#^mutually adjusted for duration in the same regression model; and * statistically significant at *p* ≤ 0.05.

Abbreviations: aOR: adjusted odds ratio; CI: confidence interval.

### 3.2 Stratification Analysis Based on Income Status, Maternal Age, and Maternal Educational Levels

Among poor families (Poorest and Poorer) in sub‐Saharan countries, pregnant women who did not take iron supplements consistently showed trends toward increased odds of having an LBW infant (aOR 1.36; 95% CI: 1.19, 1.55; *p* < 0.001), with consistent trends in both low‐and lower‐middle‐income countries. Taking iron supplements for at least 90 days remained the same with lower LBW odds in all sub‐Saharan countries (aOR 0.74; 95% CI: 0.64, 0.84; *p* < 0.001), and this finding was consistent in low‐ and lower‐middle‐income countries (Table 3). When stratified by income status, among high‐income families (richer and richest), taking supplementation for at least 90 days did not show statistically significant reductions in LBW odds (all: aOR 0.89; 95% CI: 0.77, 1.05; *p* = 0.11, low income: aOR 0.91; 95% CI: 0.74, 1.12; *p* = 0.37, lower‐middle income: aOR 0.85; 95% CI: 0.66, 1.08; *p* = 0.18) (Table S2).

**TABLE 3:** Stratified analysis of iron supplementation and its duration on low birth weight by income, maternal age, and maternal educational level.

Subgroup		Sub‐Saharan countries aOR (95%CI)	*p*	Low‐income aOR (95%CI]	*p*	Lower‐middle‐income aOR (95%CI)	*p*
Poor family income status (poorest and poorer) (*n* = 34,707)^a^							
Iron supplementation^#^	Yes	1	< 0.001*	1	0.04*	1	< 0.001*
	No	1.36 (1.19, 1.55)		1.21 (1.01, 1.46)		1.48 (1.21, 1.82)	
Duration of iron supplementation	≥ 90 days	0.74 (0.64, 0.84)	< 0.001*	0.82 (0.68, 0.99)	0.04*	0.67 (0.54, 0.82)	< 0.001*
	< 90 days	0.91 (0.81, 1.03)	0.14	0.93 (0.79, 1.09)	0.38	0.88 (0.73, 1.06)	0.19
	No	1		1		1	
Young age groups (15 to 24) years (*n* = 38,288)^b^							
Iron supplementation^#^	Yes	1	0.03*	1	0.32	1	0.09
	No	1.38 (1.03, 1.86)		1.22 (0.83, 1.78)		1.59 (0.94, 2.70)	
Duration of iron supplementation	≥ 90 days	0.72 (0.54, 0.97)	0.03*	0.82 (0.56, 1.20)	0.32	0.62 (0.37, 1.07)	0.09
	< 90 days	0.82 (0.62, 1.07)	0.14	0.84 (0.60, 1.18)	0.34	0.76 (0.46, 1.26)	0.28
	No	1		1		1	
Lower maternal education (no education + primary) (*n* = 59,582)^c^							
Iron supplementation^#^	Yes	1	0.01*	1	0.37	1	0.07
	No	1.14 (1.03, 1.27)		1.06 (0.93, 1.21)		1.19 (0.99, 1.44)	
Duration of iron supplementation	≥ 90 days	0.87 (0.79, 0.97)	0.01*	0.94 (0.82, 1.07)	0.37	0.84 (0.69, 1.01)	0.07
	< 90 days	1.01 (0.92, 1.12)	0.80	0.99 (0.88, 1.12)	0.92	1.06 (0.88, 1.26)	0.55
	No	1		1		1	
Higher maternal education (secondary + higher) (*n* = 34,638)^d^							
Iron supplementation^#^	Yes	1	0.01*	1	0.09	1.26	0.04*
	No	1.27 (1.06, 1.52)		1.28 (0.96, 1.73)		1.26 (1.01, 1.57)	
Duration of iron supplementation	≥ 90 days	0.79 (0.66, 0.94)	0.01*	0.78 (0.58, 1.04)	0.09	0.79 (0.64, 0.99)	0.04*
	< 90 days	1.00 (0.84, 1.19)	0.98	1.03 (0.78, 1.35)	0.84	0.98 (0.78, 1.23)	0.85
	No	1		1		1	

*Note:* Educational status of the mother^c,d^, partner's educational status, age of the mother^b^, media exposure, wealth index^a^, access to care, parity, antenatal care service utilization, and residence were controlled;^#^ mutually adjusted for duration in the same regression model; * statistically significant at *p* ≤ 0.05;^a,b,c,d^ covariates not included in each model.

Abbreviations: aOR: adjusted odds ratio; CI: confidence interval.

In sub‐Saharan and lower‐middle‐income countries, taking iron supplements for 90 days or longer among women with higher educational status (secondary and above) had significantly lower odds of having an LBW infant (aOR 0.79; 95% CI: 0.66, 0.94; *p* = 0.01 and aOR 0.79; 95% CI: 0.64, 0.99; *p* = 0.04, respectively). This association was not observed in low‐income countries. Similarly, among women with lower educational status (none or primary level) in sub‐Saharan countries, taking supplements for 90 days or longer showed significant trends toward reduced odds of LBW (aOR 0.87; 95% CI: 0.79, 0.97; *p* = 0.01) (Table 3).

Age‐stratified analysis showed that pregnant women aged 15–24 in sub‐Saharan countries who did not take iron supplements had significantly increased odds of LBW (aOR 1.38; 95% CI: 1.03, 1.86; *p* = 0.03). Taking supplements for 90 days or longer significantly reduced these odds by 28% (aOR 0.72; 95% CI: 0.54, 0.97; *p* = 0.03) (Table 3). Further analysis revealed that among young women (15–24 years) with no educational status, not taking oral iron supplementation remained consistent but with less statistical confidence (aOR 1.21; 95%CI: 0.89, 1.63; *p* = 0.31); this trend was consistent in low and lower‐middle‐income countries. Taking iron for at least 90 days did not yield statistically significant results (all: aOR 0.83; 95%CI: 0.60, 1.18; *p* = 0.31, low income: aOR 1.16; 95%CI: 0.72, 1.86; *p* = 0.55, lower‐middle income: aOR 0.68; 95%CI: 0.41, 1.14; *p* = 0.15) (Table S2). The lower proportion of women taking iron supplements for at least 90 days, 20.53% in sub‐Saharan countries, 19.45% in low‐income countries, and 21.26% in lower‐middle‐income countries, may have contributed to the nonsignificant results (Table S3).

In sub‐Saharan countries, taking iron supplements for 90 days or more significantly reduced the odds of LBW among partners with higher educational status (secondary and above) (aOR 0.83; 95% CI: 0.71,0.97; *p* = 0.02). However, this association was not observed when stratified based on the countries' income levels (Table S4). Interestingly, among women with no maternal and partner's education, taking iron supplementation for 90 days or more significantly reduced the odds of LBW (all: aOR 0.79; 95%CI: 0.67, 0.92; *p* < 0.01 and lower‐middle income: aOR 0.73; 95%CI: 0.56, 0.94; *p* = 0.01), although not in low‐income countries: aOR 0.85; 95%CI: 0.69, 1.06; *p* = 0.15 (Table S2).

## 4 Discussion

These results build on existing research surrounding the broader topic of maternal supplementation at the global level. The prevalence of low birth weight (LBW) in sub‐Saharan Africa is significant, standing at 10.36%. Similarly, adherence to iron supplementation is 37.34%, with a high variation between lower‐middle‐income countries (44.27%) and low‐income countries (27.93%). Our findings suggest that not taking iron supplementation during pregnancy increases the likelihood of LBW, but taking iron supplements for longer durations (at least 90 days) markedly decreases the odds of LBW in sub‐Saharan Africa, particularly when considering different income levels.

Taking iron supplements for 90 days or more significantly reduced the odds of LBW in sub‐Saharan countries, consistent with findings from Indonesia (Saptarini et al. 2023). This effect was not observed in those who took supplements for less than 90 days, likely due to the need for continuous supplementation throughout pregnancy. Pregnant women require increasing amounts of iron as pregnancy progresses, with daily needs ranging from 0.8 to over 6 mg (Bothwell 2000). Prolonged supplementation improves outcomes, particularly in low‐resource settings like sub‐Saharan Africa, where infectious diseases such as malaria (Desai et al. 2007) and hookworm (Brooker et al. 2008), along with widespread malnutrition and poor healthcare utilization (Abekah‐Nkrumah 2019; Desyibelew and Dadi 2019), exacerbate iron deficiency. Adherence to at least 90 days of supplementation is critical in such contexts. Moreover, this study highlighted that accounting for both supplementation and its duration intensified the observed impact of not taking supplements, emphasizing the importance of addressing these interrelated factors to reduce LBW outcomes.

Income stratification revealed that the absence of iron supplementation increases the likelihood of LBW among poor families in sub‐Saharan and lower‐middle‐income countries. Taking iron supplements for at least 90 days offers protection against LBW in these groups. This aligns with their increased susceptibility to iron deficiency due to limited dietary options and heightened pregnancy demands (Aspuru et al. 2011). However, the association was not observed in low‐income countries, where the severity of poverty likely overshadows the benefits of iron supplementation alone.

Iron supplementation for at least 90 days reduces LBW odds in young women (15–24 years old) in sub‐Saharan Africa. This might be due to their differing iron needs. Younger women have increased demands due to growth and development; all factors are often compounded by existing anemia in the region (Merid et al. 2023; Rai et al. 2022). Future research that considers pregnancy complications and socioeconomic background can further elucidate how age interacts with iron supplementation to influence LBW risk.

Our analysis revealed a critical influence of socioeconomic factors on iron supplementation's effectiveness. Among women with lower education, supplementation duration ≥ 90 days reduced LBW odds overall. Additionally, the findings also indicate that women with no education and a partner with no education represent a particularly vulnerable population, potentially due to limited healthcare access, high malnutrition, food insecurity, and lower program awareness (Mukaila et al. 2021; Nuamah et al. 2019). Conversely, well‐educated women benefited more from longer supplementation, likely due to better healthcare, improved diets, and potentially higher program adherence associated with their background (Ba et al. 2019; Lam et al. 2013). To bridge these gaps, interventions should improve healthcare services, increase health literacy, and supplementation awareness, including understanding the benefits among women with limited education. This suggests that improving maternity services in sub‐Saharan countries requires a multifaceted approach that addresses not only access to healthcare infrastructure but also educating young women on the benefits of such supplementation during pregnancy.

Furthermore, the lack of observed associations in low‐income countries highlights the complex interplay between poverty and iron deficiency. Severe poverty can restrict access to and distribution of supplements, even if programs exist. Financial constraints, logistical challenges, and limited awareness can all hinder utilization. Additionally, poverty often coincides with malnutrition, food insecurity, and a higher burden of infections, which can further complicate iron absorption and needs (Dahab and Sakellariou 2020; Essendi et al. 2015). Addressing these broader issues beyond iron supplementation is crucial, particularly for younger and uneducated mothers. Robust maternal health programs are essential to improving access to healthcare, overall nutrition, and education on healthy pregnancy practices for these vulnerable populations. Furthermore, this research highlights the need for more robust preventive measures, including policies that promote maternal nutritional health before pregnancy and ensure the provision of critical nutrients, such as iron, during pregnancy. As observed in this study, efforts to improve adherence are also necessary, as adherence to iron supplementation remains low in low‐income settings (27.93%) and is likely to be lower among women with no education and limited access.

Adherence to iron supplementation in sub‐Saharan Africa was 37.34%, a notable increase from the 28.7% reported in a 2019 study from the region (Ba et al. 2019). However, despite this progress, nearly half of the pregnant women in the region still fell short of adhering to the recommended duration of supplementation. This shortfall may be attributed to factors such as high levels of illiteracy, a lack of understanding of its importance for health and well‐being, and economic challenges. For example, only 7.31% of women aged 15 to 19 (likely due to unawareness of their pregnancy), 19.9% of women from the poorest economic backgrounds, and 28% of illiterate women adhered to iron supplementation for 90 days or more (Table S4). Therefore, developing targeted programs for less‐educated women, economically disadvantaged groups, and younger women is essential for enhancing adherence to iron supplementation.

This study's strengths include its large sample size, the use of DAGs to identify potential confounders, and the identification of targeted groups, such as women and their partners with lower education and those from poor socioeconomic backgrounds, for intervention. However, there are limitations, including the reliance on maternal recall for data collection, which may introduce bias, and the absence of information on maternal obesity, dietary intake during pregnancy (such as data collected through a 24 h dietary recall method), and pregnancy complications, which could influence the observed outcomes but are likely to observe greater effects than what was observed here. Additionally, the cross‐sectional design limits the ability to establish causality, underscoring the need for longitudinal studies to confirm these findings. Moreover, the lack of reporting on the specific brands of iron tablets provided in each country represents another limitation.

## 5 Conclusion

This study highlights the substantial impact of maternal iron supplementation on reducing LBW in sub‐Saharan Africa. While not taking iron supplementation increases the odds of LBW, taking supplements regularly for at least 90 days markedly lowers the odds, particularly among poor families, younger women, and women and their partners with no education. However, the benefits are less pronounced in low‐income countries, underscoring the need to address severe poverty, healthcare access, and nutritional deficiencies. To maximize the effectiveness of iron supplementation programs, it is crucial to ensure that policies are inclusive and address the needs of vulnerable populations. Strengthening healthcare infrastructure and improving transportation systems for pregnant women to access healthcare facilities are vital steps. Additionally, implementing educational programs to raise awareness even among young women about the importance of iron supplementation and utilizing community health workers to reach and educate women in lower socioeconomic groups may help address disparities and promote positive health behaviors.

Future research should focus on collecting comprehensive data on mothers, pregnancy, and socioeconomic data to clarify these relationships further. Longitudinal studies are also necessary to establish causality and better quantify the long‐term effects of iron supplementation on LBW.

## Author Contributions

**Yibeltal Bekele:** conceptualization (equal), formal analysis (equal), methodology (equal), writing – original draft (equal), writing – review and editing (equal). **Don Vicendese:** writing – review and editing (equal). **Melissa Buultjens:** conceptualization (equal), writing – review and editing (equal). **Mehak Batra:** conceptualization (equal), methodology (equal), writing – original draft (equal), writing – review and editing (equal). **Bircan Erbas:** conceptualization (equal), methodology (equal), writing – review and editing (equal).

## Conflicts of Interest

The authors declare no conflicts of interest.

## Supporting information


Data S1.

## Data Availability

Data is available upon request on The DHS program websiteat: https://www.dhsprogram.com/data/available‐datasets.cfm.
