# Supplementary material for: Do Maternal Factors Modify the Associations Between Iron Supplementation and Low Birth Weight in Sub‐Saharan Africa?
Source: Food Sci Nutr. 2025 Apr 3;13(4):e70078. doi: 10.1002/fsn3.70078 (PMC11968328; doi:10.1002/fsn3.70078)
Supplement: Supplementary file 1 — Data S1. [file FSN3-13-e70078-s001.docx]

**Supplementary appendix**

**Method supplementary file**

Information on the confounding variables was collected via maternal recall. These included antenatal care utilisation (yes or no) with the question, “Did you see anyone for antenatal care for this pregnancy?”, maternal age at birth of offspring (categorised as 15 to 19, 20 to 24, 25 to 29, 30 to 34, 35 to 39, 40 to 44 or 45 to 49), mother’s educational status (illiterate “not attained education”, primary “grade 1 to 8”, secondary “grade 9 to 12” or higher “college and university”), partner's educational level (illiterate “not attained education”, primary “grade 1 to 8”, secondary “grade 9 to 12” or higher “college and university”), healthcare access (a big problem or not a big problem), parity (nullipara “not given birth”, primipara ‘one birth”, multipara “2 to 4 births” or grand multipara “five or more”), wealth index (poorest, poorer, middle, richer or richest), media exposure(Television or radio or newspaper) (yes or no), marital status (single, married, separated, divorced or widowed ), and maternal occupation (yes or no).


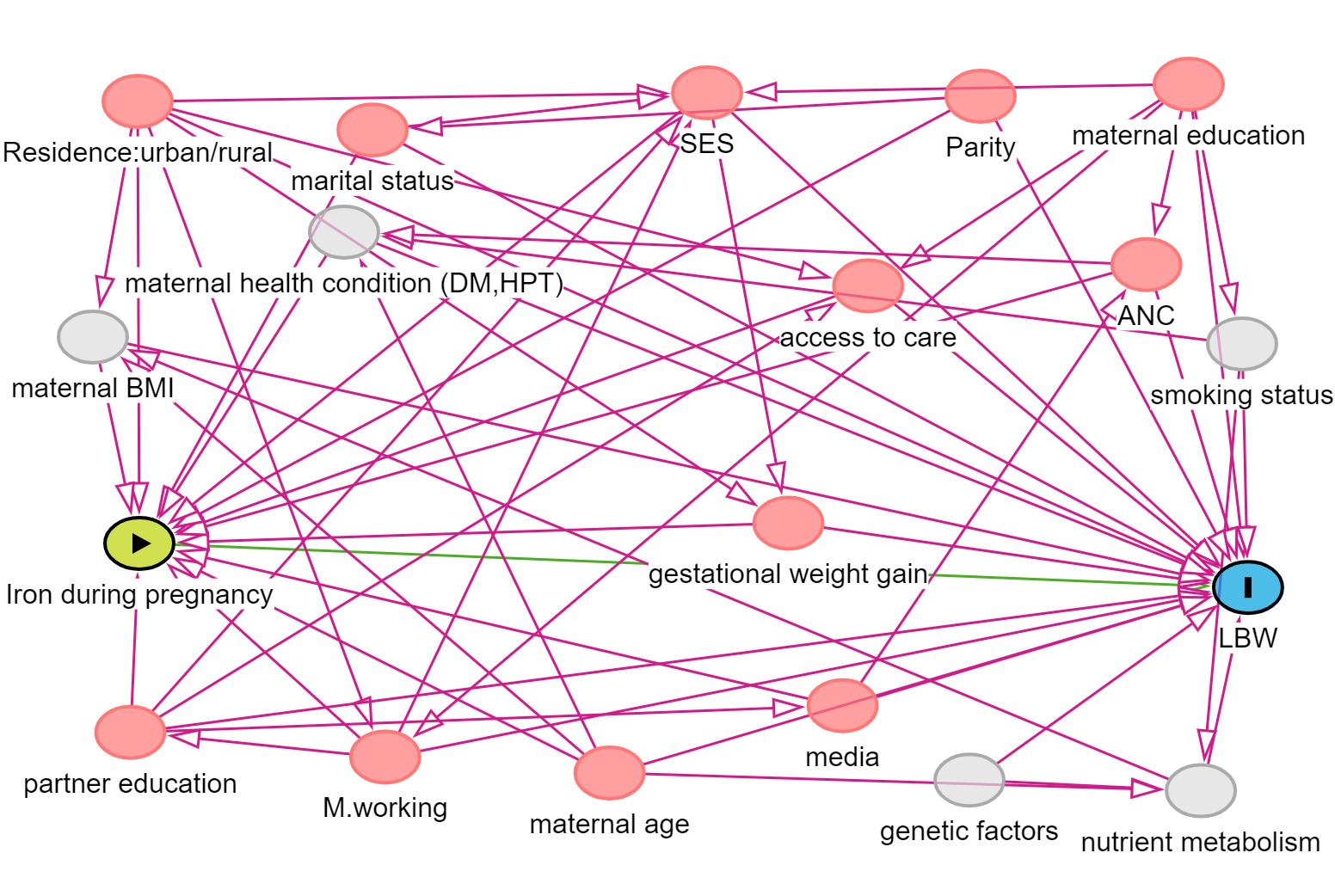


Supplementary Figure 1. Direct acyclic graph used to understand the causal pathways: ANC: antenatal care, SES: Socio-Economic Status, maternal BMI: Maternal Body Mass Index, M.working: Maternal Working Status, LBW: Low Birth Weight, DM: Diabetes Mellitus and HPT: Hypertension.

Supplementary Table 1: Crude association of iron supplementation and its duration on low birth weight in sub-Saharan countries.

| Exposures | | sub-Saharan countries  cOR (95%CI) | P-value | Low-income  cOR (95%CI) | P-value | Lower-middle-income  cOR (95%CI) | P-value |
| --- | --- | --- | --- | --- | --- | --- | --- |
| Iron supplementation | Yes | 1 | **<0.001*** | 1 | 0.09 | 1 | **<0.001*** |
|  | No | **1.11 (1.04, 1.18)** |  | 1.09 (0.98, 1.21) |  | **1.09 (1.01, 1.25)** |  |
| Duration of iron supplementation | ≥90 days | **0.83 (0.77, 0.89)** | **<0.001*** | **0.85 (0.76, 0.96)** | **0.01*** | **0.82 (0.71, 0.94)** | **0.01*** |
|  | <90 days | 0.97 (0.90,1.04) | 0.38 | 0.97 (0.87, 1.08) | 0.57 | 1.03 (0.89, 1.18) | 0.70 |
|  | No | 1 |  | 1 |  | 1 |  |

Footnotes: cOR: crude odds ratio, CI: confidence interval, * statistically significant at p-value ≤ 0.05

Supplementary Table 2: Stratification analysis by family income, maternal age, and educational status of women and partners in sub-Saharan countries.

| Subgroup | | sub-Saharan countries  aOR (95%CI) | P-value | Low-income  aOR (95%CI]) | P-value | Lower-middle-income  aOR (95%CI) | P-value |
| --- | --- | --- | --- | --- | --- | --- | --- |
| **High family income status (richer and richest) (n=40,526) ^a^** | | | | | | | |
| Iron supplementation ^#^ | Yes | 1 | 0.18 | 1 | 0.37 | 1 | 0.18 |
|  | No | 1.11 (0.95, 1.30) |  | 1.10 (0.90, 1.35) |  | 1.18 (0.92, 1.50) |  |
| Duration of iron supplementation | ≥90 days | 0.89 (0.77, 1.05) | 0.18 | 0.91 (0.74, 1.12) | 0.37 | 0.85 (0.66, 1.08) | 0.18 |
|  | <90 days | 1.11 (0.96, 1.29) | 0.14 | 1.08 (0.89, 1.30) | 0.45 | 1.14 (0.89, 1.46) | 0.29 |
|  | No | 1 |  | 1 |  | 1 |  |
| **Higher partner educational status (secondary and above) (n=40,951) ^b^** | | | | | | | |
| Iron supplementation ^#^ | Yes | **1** | **0.015*** | 1 | 0.11 | 1 | 0.09 |
|  | No | **1.20 (1.02, 1.41)** |  | 1.24 (0.97, 1.60) |  | 1.18 (0.96, 1.44) |  |
| Duration of iron supplementation | ≥90 days | **0.83 (0.71, 0.97)** | **0.02*** | 0.81 (0.64, 1.05) | 0.11 | 0.84 (0.69, 1.03) | 0.09 |
|  | <90 days | 1.02 (0.87, 1.20) | 0.79 | 0.98 (0.76, 1.25) | 0.86 | 1.06 (0.85, 1.31) | 0.61 |
|  | No | 1 |  | 1 |  | 1 |  |
| **Young age groups (15 to 24) years and no educational status (n=5419) ^c^** | | | | | | | |
| Iron supplementation ^#^ | Yes | 1 | 0.31 | 1 | 0.55 | 1 | 0.15 |
|  | No | 1.21 (0.89, 1.63) |  | 0.99 (0.87, 1.39) |  | 1.47 (0.87, 2.46) |  |
| Duration of iron supplementation | ≥90 days | 0.83 (0.60, 1.18) | 0.31 | 1.16 (0.72, 1.86) | 0.55 | 0.68 (0.41, 1.14) | 0.15 |
|  | <90 days | 0.86 (0.62, 1.19) | 0.38 | 0.93 (0.60, 1.44) | 0.74 | 0.89 (0.55, 1.44) | 0.64 |
|  | No | 1 |  | 1 |  | 1 |  |
| **No partner education and no maternal education (n=34,638) ^d^** | | | | | | | |
| Iron supplementation ^#^ | Yes | 1 | **0.01*** | 1 | 0.11 | **1** | **0.01*** |
|  | No | **1.24 (1.04, 1.49)** |  | 1.23 (0.96, 1.59) |  | **1.24 (1.05, 1.68)** |  |
| Duration of iron supplementation | ≥90 days | **0.79 (0.67, 0.92)** | **0.01*** | 0.85 (0.69, 1.06) | 0.11 | **0.73 (0.56, 0.94)** | **0.01*** |
|  | <90 days | 0.94 (0.81, 1.09) | 0.40 | 0.93 (0.76, 1.12) | 0.44 | 0.95 (0.75, 1.23) | 0.72 |
|  | No | 1 |  | 1 |  | 1 |  |

**Footnote:** Educational status of the mother ^c, d^, partner's educational status **^b, d^**, age of the mother, media exposure ^c^, wealth index ^a^, access to care, parity, antenatal care service utilisation, and residence were controlled; ^#^mutually adjusted for duration in the same regression model, aOR: adjusted odds ratio, CI: confidence interval, * statistically significant at p-value ≤ 0.05, ^a, b, c, d^ covariates not included in each model.

Supplementary Table 3: Descriptive characteristics of the participants with iron supplementation and its duration no maternal education with maternal age 15 to 24 years and no education in sub-Saharan countries, 2024.

| Maternal age 15 to 24 years and no maternal education | | | | | | | |
| --- | --- | --- | --- | --- | --- | --- | --- |
| Subgroup | | sub-Saharan countries | | Low-income | | Lower-middle-income | |
|  |  | Age 15-24 & no education | p-value | Age 15-24 & no education | p-value | Age 15-24 & no education | p-value |
| Iron supplementation  n=14999 | Yes | 10,464 (21.56) | <0.001* | 4440 (19.86) | <0.001* | 6024 (23.02) | <0.001* |
|  | No | 4535 (23.53) |  | 1680(20.54) |  | 2855 (25.73) |  |
| Duration of iron supplementation  n=14,254 | No | 4535(23.53) | <0.001* | 1680 (20.54) | <0.001* | 2855(25.73) | <0.001* |
|  | <90 days | 5897(22.55) |  | 2547 (20.01) |  | 3350(24.96) |  |
|  | ≥ 90 days | 3822 (20.53) |  | 1461 (19.45) |  | 2361 (21.26) |  |
| No partner education and no maternal education | | | | | | | |
| Exposures | | sub-Saharan countries | | Low-income | | Lower-middle-income | |
|  |  | No partner & maternal education | p-value | No partner & maternal education | p-value | No partner & maternal education | p-value |
| Iron supplementation.  n=43,367 | Yes | 30,460 (68.18) | <0.001 | 14,079(68.70) | <0.001 | 16,381 (67.73) | <0.001 |
|  | No | 12,907 (73.65) |  | 4933 (66.97) |  | 7974 (78.49) |  |
| Duration of iron supplementation (40,908) | No | 12,907(73.65) | <0.001 | 4933(66.97) | <0.001 | 7974 (78.49) | <0.001 |
|  | <90 days | 16,360 (68.16) |  | 7720 (66.56) |  | 8640(69.64) |  |
|  | ≥ 90 days | 11,641 (67.48) |  | 4909 (70.55) |  | 6732 (65.41) |  |

Supplementary Table 4: Descriptive characteristics of the participants with iron supplementation and its duration in sub-Saharan countries, 2024.

| Sub-Saharan countries | | | | | | |
| --- | --- | --- | --- | --- | --- | --- |
| Exposures | | Iron supplementation | | Duration of the supplementation | | |
|  |  | Yes (%) | No (%) | No | <90 days | ≥ 90 days |
| Age *  n=188,990 | 15-19 | 11,577 (7.62) | 2863 (7.71) | 2863(7.71) | 5906(7.86) | 4829(7.31) |
|  | 20-24 | 34,940 (23.01) | 8009 (21.58) | 8009(21.58) | 17,598(23.41) | 15,081(22.84) |
|  | 25-29 | 38,115 (25.10) | 8,982 (24.20) | 8982(24.20) | 18,511(24.64) | 16,936(25.65) |
|  | 30-34 | 30,748 (20.24) | 7434 (20.03) | 7434(20.03) | 14,996(19.96) | 13.586(20.57) |
|  | 35-39 | 22,580 (14.87) | 5664 (15.26) | 5664 (15.26) | 11,073(14.74) | 9825(14.88) |
|  | 40-44 | 10,580 (14.87) | 3015 (8.12) | 3015(8.12) | 5302(7.06) | 4454(6.75) |
|  | 45-49 | 3340 (2.20) | 1143 (3.08) | 1143(3.08) | 1754(2.33) | 1322(2.00) |
| Educational status*  n=188,990 | Illiterate | 48,525 (31.95) | 19,276 (51.94) | 19,276(51.94) | 26,149 (34.80) | 18,614 (28.19) |
|  | Primary | 49,703(32.73) | 11,175(30.11) | 11,175(30.11) | 27,165 (36.16) | 19,888(30.12) |
|  | Secondary | 45,853(30.19) | 5883(15.85) | 5883 (15.85) | 19,143 (25.48) | 22,992(34.82) |
|  | Higher | 7799(5.13) | 776 (2.09) | 776 (2.09) | 2674 (3.56) | 4539(6.87) |
| Wealth index*  n=188,990 | Poorest | 33476 (22.04) | 13,168 (35.48) | 13,168(35.48) | 18,272(24.32) | 13,141(19.90) |
|  | Poorer | 31717 (20.88) | 8783 (23.67) | 8783 (23.67) | 16,655 (22.17) | 12,972 (19.64) |
|  | Middle | 31,248(20.57) | 6483 (17.47) | 6483(17.47) | 15,439 (20.55) | 13,700(20.75) |
|  | Richer | 29,240 (19.25) | 4772 (12.86) | 4772(12.86) | 13,667(18.19) | 13,301(20.14) |
|  | Richest | 26,196 (17.25) | 3904 (10.52) | 3904 (10.52) | 11,098(14.77) | 12,919(19.56) |
| Maternal working*  n=188,990 | Yes | 95,485 (62.87) | 22,354 (60.24) | 22,354(60.24) | 48,401(64.42) | 40,843 (61.85) |
|  | No | 56,395 (37.13) | 14,756 (39.76) | 14,756(39.76) | 26,730 (35.58) | 25,190 (38.15) |
| ANC*  N=188,990 | Yes | 149,505 (98.44) | 21,130 (56.94) | 21,130(56.94) | 73,468(97.79) | 65,539(99.25) |
|  | No | 2375 (1.56) | 15,980 (43.06) | 15980 (43.06) | 1663 (2.21) | 494(0.75) |
| Parity*  n=188,990 | Nullipara | 34463 (22.69) | 6808 (18.35) | 6808 (18.35) | 16,166(21.52) | 15,841(23.99) |
|  | Primipara | 30,098 (19.82) | 6,518 (17.56) | 6518 (17.56) | 14,463 (19.25) | 13,559(20.53) |
|  | Multipara | 59,430 (39.13) | 14422 (38.86) | 14,422(38.86) | 29,622(39.43) | 25,598 (38.77) |
|  | Grand multipara | 27,889 (18.36) | 9,362 (25.23) | 9362 (25.23) | 14,880 (19.81) | 11,033(16.71) |
| Partner education*  n=158,222 | Illiterate | 40,718 (32.18) | 15,298 (48.28) | 15,298(48.25) | 21,512(34.04) | 15,929(29.14) |
|  | Primary | 35,542 (28.09) | 9271 (29.24) | 9271 (29.24) | 20,240 (32.02) | 13,673 (25.02) |
|  | Secondary | 38,263 (30.24) | 5777 (18.22) | 5777 (18.22) | 16,870 (26.69) | 18,598(34.03) |
|  | Higher | 11,991 (9.48) | 1362 (4.30) | 1362 (4.30) | 4580 (7.25) | 6455(11.81) |

Footnotes: chi-square test was used, ANC: antenatal care. * p-value = <0.001.
